# Neuroprotective Effect of a Novel ATP-Synthase Inhibitor Bedaquiline in Cerebral Ischemia-Reperfusion Injury

**DOI:** 10.3390/ijms22189717

**Published:** 2021-09-08

**Authors:** Danielius Umbrasas, Odeta Arandarcikaite, Ramune Grigaleviciute, Rimantas Stakauskas, Vilmante Borutaite

**Affiliations:** 1 Neuroscience Institute, Lithuanian University of Health Sciences, LT-47181 Kaunas, Lithuania; odeta.arandarcikaite2@lsmuni.lt (O.A.); vilmante.borutaite@lsmuni.lt (V.B.); 2 Biological Research Center, Lithuanian University of Health Sciences, LT-47181 Kaunas, Lithuania; ramune.grigaleviciute@lsmuni.lt (R.G.); rimantas.stakauskas@lsmuni.lt (R.S.)

**Keywords:** mitochondrial respiration, bedaquiline, stroke, ischemia, neuroprotection, ATP synthase

## Abstract

Mitochondrial dysfunction during ischemic stroke ultimately manifests as ATP depletion. Mitochondrial ATP synthase upon loss of mitochondrial membrane potential during ischemia rapidly hydrolyses ATP and thus contributes to ATP depletion. Increasing evidence suggests that inhibition of ATP synthase limits ATP depletion and is protective against ischemic tissue damage. Bedaquiline (BDQ) is an anti-microbial agent, approved for clinical use, that inhibits ATP synthase of Mycobacteria; however recently it has been shown to act on mitochondrial ATP synthase, inhibiting both ATP synthesis and hydrolysis in low micromolar concentrations. In this study, we investigated whether preconditioning with BDQ can alleviate ischemia/reperfusion-induced brain injury in Wistar rats after middle cerebral artery occlusion-reperfusion and whether it affects mitochondrial functions. We found that BDQ was effective in limiting necrosis and neurological dysfunction during ischemia-reperfusion. BDQ also caused inhibition of ATPase activity, mild uncoupling of respiration, and stimulated mitochondrial respiration both in healthy and ischemic mitochondria. Mitochondrial calcium retention capacity was unaffected by BDQ preconditioning. We concluded that BDQ has neuroprotective properties associated with its action on mitochondrial respiration and ATPase activity.

## 1. Introduction

Stroke currently is one of the leading causes of disability and death in adults in developed countries [1]. Numerous forms and subtypes of stroke are recognized but generally, it can be classified into two categories: hemorrhagic stroke and ischemic stroke, the latter of which accounts for about 70% of all stroke cases worldwide [2]. The treatment of ischemic stroke focuses on restoring blood supply to the affected tissue either pharmacologically with recombinant tissue plasminogen activator; or surgically via endovascular thrombectomy [3]. These treatments are highly effective in reducing mortality after stroke; however, not all stroke patients are eligible for thrombolysis or thrombectomy [4,5]. Only a small portion of patients can undergo such procedures because of the risks associated with these treatments: cerebral hemorrhage in the case of thrombolysis or procedure-associated complications in the case of thrombectomy [6,7]. Due to the limitations of currently available ischemic stroke treatments, in recent years there has been a vast increase in studies investigating new neuroprotective strategies that focus more on the damaged brain tissue than the vascular component of stroke. As mentioned before, restoration of blood supply to the affected brain region is very important to prevent death and limit the development of disability, but this restoration of blood flow causes a phenomenon known as “reperfusion injury” [8,9]. During reperfusion, there is a significant increase in reactive oxygen species (ROS), accumulation of leukocytes and platelets in the infarcted area, activation of the complement system, and changes in mitochondrial signalling [10].

Mitochondria play an important role in the pathogenesis of ischemic stroke, as they provide brain cells with most of the ATP and are major regulators of cell death [11]. In ischemic conditions, the lack of oxygen and glucose inhibits mitochondrial respiration and in turn, ATP synthesis, which leads to energy depletion and eventually necrotic cell death [12]. Impaired work of the mitochondrial electron transport chain (ETC) results in the collapse of the electrochemical proton gradient across the inner mitochondrial membrane (IMM). Under such conditions, F1F0-ATP synthase (further referred to as ATPase) starts to hydrolyse ATP to maintain mitochondrial membrane potential (Δψ_m_), and this contributes even more to the loss of ATP energy during ischemia [13]. ATPase is an enzyme complex located in the IMM. The main function of this complex under physiological conditions is phosphorylation of ADP to make ATP while utilizing the electrochemical gradient created by the mitochondrial ETC. Interestingly, from a thermodynamic point of view, cellular conditions favour ATP hydrolysis reaction over synthesis; however, ATPase is able to harness an external force (the electrochemical proton gradient) to keep the reaction going against thermodynamic equilibrium [14]. During ischemia, the ETC complexes are damaged; therefore, Δψ_m_ along with the proton motive force, severely decreases [15,16]. ATPase no longer has the external force to drive ATP synthesis, and as a result, rapidly hydrolyses ATP while pumping protons out of the mitochondrial matrix in an effort to maintain Δψ_m_ [17]. Besides ATP synthesis and maintaining IMM potential, ATPase is also involved in the regulation of cell death, formation of mitochondrial cristae, and permeability transition [18,19,20,21]. Because of the central role of this enzyme in energy metabolism as well as involvement in other important cellular processes, ATPase has been suggested as a potential therapeutic target for a plethora of diseases including heart failure, neurodegenerative diseases, cancer, and microbial infections [22].

It has been suggested that ATPase plays a role in ischemic preconditioning in the heart [23] and the brain [24]. The main factor regulating ATPase activity during preconditioning is suggested to be a protein called mitochondrial F0F1 ATPase inhibitory factor 1 (IF1), which inhibits ATP hydrolysis by ATPase [25]. This protein seems to be expressed in response to hypoxia-inducible factor 1α (HIF-1α) in liver and brain cells [24,26]. Experimental overexpression of IF1 was shown to have a neuroprotective effect for neurons after oxygen-glucose deprivation (OGD), and IF1 knockdown neurons showed a higher rate of ATP depletion during periods of OGD, suggesting that inhibition of ATPase during ischemia leads to the preservation of ATP and reduced neuronal cell death [24]. Based on this research, we hypothesized that pharmacological inhibition of ATPase may result in a similar effect and reduce ischemic damage after middle cerebral artery occlusion (MCAO)/reperfusion.

Mitochondrial permeability transition pore (mPTP) is suggested to be one of the pathogenetic mechanisms of ischemia-reperfusion injury. mPTP is a structure inside the inner mitochondrial membrane (IMM) that is permeable to ions and solutes up to 1.5 kDa [27]. This non-specific pore is activated by high matrix Ca^2+^ concentration, and prolonged opening of mPTP leads to mitochondrial swelling, loss of Δψ_m_, cessation of ATP synthesis, and release of cytochrome c and other pro-apoptotic signals into the cytosol [28]. The main regulating factor of mPTP opening is matrix Ca^2+^ concentration [29], and not many endogenous molecules have been shown to regulate mPTP. However, inhibition of complex I with rotenone was shown to significantly increase the Ca^2+^ concentration at which mPTP opened [30]; therefore, complex I may be considered as a regulatory factor of mPTP opening. The molecular identity of mPTP is not entirely understood; however, there is evidence that ATPase may be involved in the formation of mPTP or its regulation [31,32,33].

Bedaquiline (BDQ) is a diarylquinoline compound that was granted accelerated approval by the Food and Drug Administration (FDA) in 2012 to be used as part of combination therapy for multidrug-resistant tuberculosis [34]. BDQ targets mycobacterial ATPase, binds to its c subunit in the F0 domain, and prevents rotation of the c-ring, thus inhibiting the enzyme complex [35]. As a result, ATP synthesis is compromised and mycobacteria undergo cell death [36]. Initially, BDQ was reported to be selective for mycobacterial ATPase with minimal or no inhibitory effect on mammalian enzyme [37,38]. However in 2020 Luo et al. published a comprehensive study on the interaction between BDQ and yeast as well as human ATPase [39]. They showed that BDQ inhibits yeast and human ATPase working both in ATP synthesis and ATP hydrolysis directions. They suggested that the binding site of BDQ is at the interface of c and a subunits in the F0 domain, which is similar to the binding site on mycobacterial ATP synthase [35]. Targeting the c ring by other compounds (e.g., oligomycin) has been shown to inhibit apoptosis [40] and protect against myocardial ischemia/reperfusion injury [41]. Therefore, we investigated whether a similar effect can be achieved by BDQ in an experimental cerebral ischemia/reperfusion model.

In this study, we aimed to investigate whether BDQ is capable of neuroprotection in the context of ischemic stroke. We hypothesize that BDQ may have two neuroprotective mechanisms: inhibition of ATP hydrolysis by mitochondrial ATP synthase (ATPase activity) and possibly inhibition of mPTP, as some studies claim that the c-ring of the F0 domain in ATP synthase is directly involved in the formation of mPTP (see review [42]). We studied the effects of pretreatment with BDQ on ischemia-damaged and healthy brain mitochondria, mainly focusing on the hydrolytic activity of ATP synthase, mitochondrial respiration, and mPTP opening.

## 2. Results

### 2.1. Effect of BDQ Treatment on the Infarct Size and Neurological Impairment of Ischemic Rats

**Figure 1:**
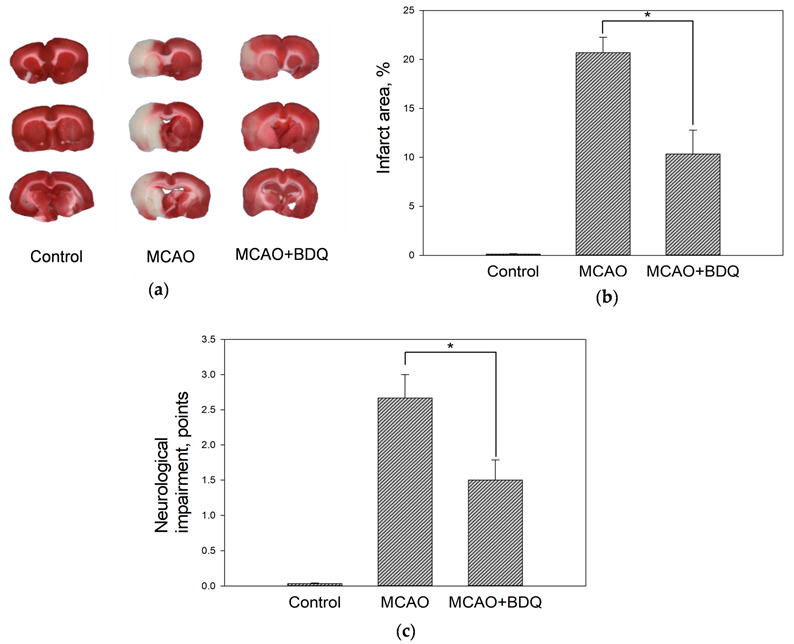
The effect of bedaquiline (BDQ) treatment on infarct size and neurological impairment. (**a**) Representative images of 2, 3, 5 triphenyltetrazolium chloride (TTC) staining of brain slices. (**b**) The effect of 60 min middle cerebral artery occlusion (MCAO)/24 h reperfusion as well as BDQ treatment on the infarct size in TTC stained brain slices. (**c**) The effect of 60 min MCAO/24 h reperfusion as well as BDQ treatment on the neurological state of the animals. Data are presented as Mean ± SEM of 3–4 experiments on individual animals. *—Statistically significant difference (*p* ≤ 0.05).

First, we evaluated whether intraperitoneal administration of BDQ has an effect on ischemia-induced brain damage by measuring infarct sizes in brains of rats subjected to MCAO. As can be seen in Figure 1a,b, the infarct size in MCAO group brains was 20.7 ± 1.6% and in the MCAO+BDQ group, it was substantially reduced, to 10.3 ± 2.4%.

We also evaluated neurological impairment in MCAO rats and found that the average score in the MCAO group was 2.6 ± 0.3 points, while in the MCAO+BDQ group, the average score was significantly reduced to 1.5 ± 0.2 points (Figure 1c). These data suggest that pretreatment with BDQ was able to reduce the neurological impairment as well as the size of brain injury after MCAO surgery.

### 2.2. Effect of MCAO and BDQ Treatment on Mitochondrial Respiration

**Figure 2:**
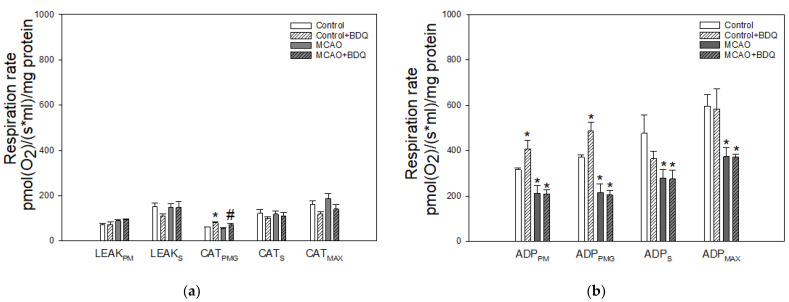

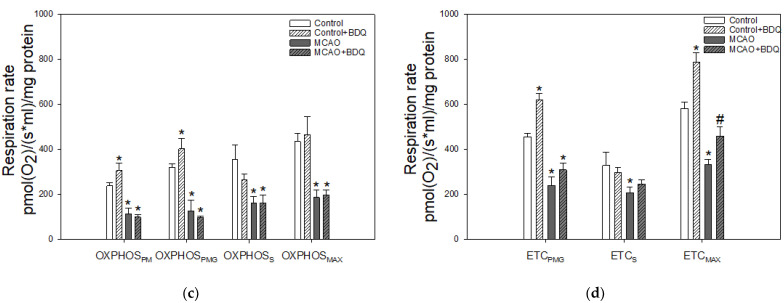
Mitochondrial respiration rates after MCAO and BDQ treatment. (**a**)—Oxygen consumption rates (OCRs) in the proton leak linked respiration states with complex I (LEAK_PM_ and CAT_PMG_), complex II (LEAK_S_ and CAT_S_), and complex I +II (CAT_MAX_) substrates; (**b**)—OCRs in the ADP-stimulated respiration states with complex I (ADP_PM_ and ADP_PMG_), complex II (ADP_S_), and complex I +II (ADP_MAX_) substrates; (**c**)—calculated values of OCRs linked to oxidative phosphorylation (identical metabolic states to Figure 3b; (**d**)—OCRs linked to maximal electron transport chain (ETC) capacities with complex I (ETC_PMG_), complex II (ETC_S_), and complex I +II (ETC_MAX_) substrates. LEAK refers to a metabolic state in which mitochondria and substrates are added to the reaction chamber without ADP; CAT refers to a metabolic state induced by the addition of 2 μM carboxyatractyloside (CAT); ADP refers to a metabolic state in which mitochondria, substrates, and ADP are present; OXPHOS refers to a calculated value representing OCRs strictly linked to oxidative phosphorylation; ETC refers to the OCRs of uncoupled mitochondria. The substrates are abbreviated: P—pyruvate; M—malate; G—glutamate; S—succinate; MAX—all of the listed substrates. Data are presented as Mean ± SEM of 6–7 experiments on individual animals. Statistically significant difference (*p* < 0.05): *—compared to control; #—compared to MCAO.

Next, we investigated whether pretreatment with BDQ affects mitochondrial respiration in healthy and ischemic brains by using high-resolution respirometry. We used freshly isolated mitochondria to evaluate oxygen consumption rates (OCRs) in different metabolic states. Figure 2a represents OCRs in proton leak-linked metabolic states: there was a tendency of increased rates in LEAK_PM_ after MCAO, but it was not further affected by BDQ treatment. No significant changes in rates of LEAK_S_ as well as CAT_S_ and CAT_MAX_ were observed after MCAO or BDQ treatment; however, in CAT_PMG_ state, the respiratory rates were increased by BDQ treatment both in control and MCAO groups (Figure 2a). These data suggest that BDQ exhibits a slight uncoupling effect in mitochondria respiring on complex I substrates in the presence of carboxyatractyloside (CAT).

Figure 2b shows OCRs in ADP-stimulated respiration states. As can be seen, BDQ pretreatment had a stimulating effect on normal mitochondria ADP-stimulated rates with pyruvate plus malate (PM) and pyruvate plus malate plus glutamate (PMG), but not with succinate (S) and pyruvate plus malate plus glutamate plus succinate (PMGS) as substrates. In the MCAO group, ADP-stimulated rates with all substrates investigated (PM, PMG, S, and PMGS) were substantially decreased by about 37%, 43%, 41%, and 37% respectively, compared to control; however, BDQ pretreatment before occlusion had no effect on ADP-stimulated rates with all substrates investigated. To obtain better insight into BDQ effects on phosphorylating respiration of mitochondria, we performed calculations subtracting the proton leak respiration rate value from the respective ADP-stimulated respiration rate value and expressed this as OXPHOS (phosphorylating respiration) rates (Figure 2c). As in the ADP states, we observed a significant effect of MCAO on OXPHOS rates with all substrates investigated; however, no significant effect of BDQ in the ischemic mitochondria was detected. BDQ treatment of normal, healthy rats resulted in higher OXPHOS_PM_ and OXPHOS_PMG_ rates compared to control. These data suggest that BDQ did not prevent ischemia-induced decrease in phosphorylating respiration of brain mitochondria, but it stimulated respiration in control mitochondria respiring on complex I substrates.

Results represented in Figure 2d show the effect of MCAO and BDQ treatment on OCRs linked to maximal ETC capacities. MCAO significantly reduced respiration in ETC_PMG_, ETC_S_, and ETC_MAX_ states by 47%, 37%, and 43%, respectively, while BDQ protected against ischemia-induced decrease in ETC_MAX_ by 38%. The positive, stimulating effect of BDQ was also observed in normal, healthy mitochondria, where BDQ significantly increased OCRs in ETC_PMG_ and ETC_MAX_ metabolic states both by 36%. These data pointed to a tendency of BDQ to stimulate the activity of respiratory complexes both in control and in ischemic mitochondria. While a statistically significant difference in ischemic groups was found only when all substrates were used (ETC_MAX_), overall data from respirometry suggest that this stimulating effect was most pronounced for complex I.

### 2.3. BDQ Effects on the Activity of ATPase

**Figure 3:**
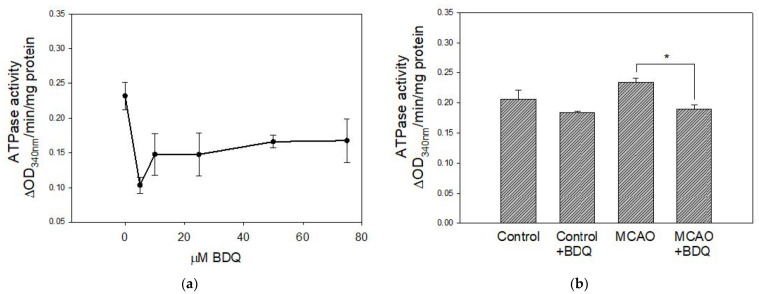
Effect of BDQ on ATPase activity. (**a**)—The direct effect of BDQ on ATPase activity in isolated normal brain mitochondria. BDQ in all concentrations significantly decreased ATPase activity (*p* < 0.05). The extent of inhibition between different concentrations of BDQ did not differ significantly (*p* ≥ 0.05). (**b**)—The effect of BDQ, injected into rats prior to MCAO or 24 h before euthanasia, on the activity of ATPase in isolated mitochondria. Data are presented as mean ± SEM of 3–6 experiments on individual animals. *—Statistically significant difference (*p* < 0.05).

BDQ is known as an inhibitor of ATP synthase in liver mitochondria [43]; however, we did not observe a BDQ inhibitory effect on OXPHOS respiration rates in brain mitochondria. Therefore, we questioned whether BDQ could inhibit a reverse reaction—hydrolysis of ATP by ATPase of isolated brain mitochondria. The direct effect of BDQ on ATPase activity in isolated brain mitochondria is represented in Figure 3a: ATPase was inhibited by about 50% with 5μM BDQ and stayed at a similar level with higher concentrations of BDQ. Pretreatment with 2 mg/kg BDQ before MCAO also resulted in a significant decrease in the activity of ATPase: in ischemic mitochondria pretreated with BDQ, ATPase activity was 19% lower compared to the MCAO group (Figure 3b). A tendency toward decreased ATPase activity was also observed in mitochondria isolated from brains of BDQ-treated healthy rats compared to control, although the effect was not statistically significant (Figure 3b). It is worth mentioning that the calculated theoretical plasma concentration of BDQ would be about 50 µM after injection of 2 mg/kg dose (assuming 100% bioavailability) and, as BDQ has been reported to be able to accumulate in CNS in rodents [44], estimated BDQ concentration in the CNS after such dose would be within the 5–75 µM range. This is consistent with the BDQ concentration range directly causing ATPase inhibition in isolated mitochondria (Figure 3a).

### 2.4. Effect of BDQ on Mitochondrial Calcium Retention Capacity

**Figure 4:**
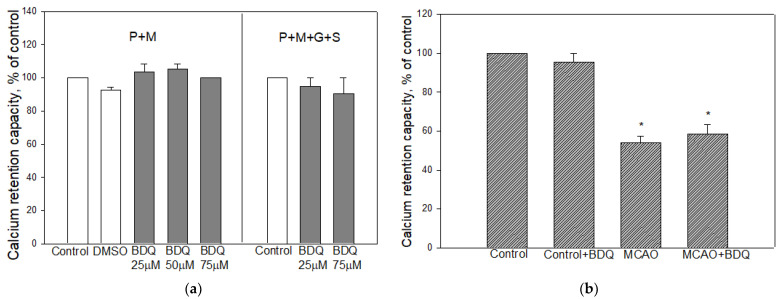
Effect of BDQ on mitochondrial calcium retention capacity. (**a**)—the direct effect of BDQ and vehicle dimethyl sulfoxide (DMSO) on isolated brain mitochondrial calcium retention capacity using pyruvate and malate or pyruvate, malate, glutamate, and succinate as substrates; (**b**)—mitochondrial calcium retention capacity after MCAO with/without BDQ using pyruvate and malate as substrates. Data are presented as Mean ± SEM of 3–6 experiments on individual animals. *—Statistically significant difference compared to control (*p* < 0.05).

To evaluate the direct effect of BDQ on mPTP opening, we measured the calcium retention capacity of mitochondria isolated from normal rat brains. These experiments were performed with BDQ dissolved in DMSO and using mixtures of substrates: PM or PMGS (Figure 4a). As can be seen in Figure 4a, 25– 75µM BDQ and vehicle DMSO had no effect on mitochondrial calcium retention capacity. We also investigated whether pretreatment with BDQ (2 mg/kg) for 24 h affects calcium retention capacity of normal and ischemia-damaged brain mitochondria. As shown in Figure 4b, mitochondrial calcium retention capacity was 127.9 ± 9.0 nmol/mg in the control group, while in the MCAO group, it was reduced by 46%. However, pretreatment with BDQ had no statistically significant effect compared to non-treated groups.

## 3. Discussion

In the present study, we for the first time demonstrated that BDQ, when administered before the onset of MCAO, exerts a neuroprotective effect against ischemia-reperfusion induced brain damage and neurological dysfunction. The results show that MCAO rats pretreated with BDQ exhibited decreased mitochondrial ATPase activity and increased proton leak-associated and uncoupled respiration rates, whereas mitochondrial calcium retention capacity remained unchanged. Direct effects of BDQ investigated on isolated brain mitochondria confirmed that BDQ inhibits mammalian ATPase but does not prevent the calcium-induced opening of the mPTP. This allows us to suggest that the neuroprotective effect of BDQ may be at least partially due to limiting ATP hydrolysis by ATPase and limiting the ischemia-induced damage to complexes of the ETC.

Inhibition of ATPase is considered to be one of the mechanisms of ischemic preconditioning [20,45]. It has been shown that neurons overexpressing the endogenous ATPase inhibitor IF1 are more resistant to cell death caused by oxygen-glucose deprivation [24] and excitotoxicity [20]. Therefore, we investigated whether pharmacological preconditioning with BDQ could lead to a similar outcome in vivo. Our results revealed that BDQ caused inhibition of ATPase that persisted for the whole reperfusion period and administration of BDQ was effective in limiting necrosis caused by ischemia-reperfusion, as was apparent from the TTC-stained brain slices.

Our results, concerning the effect of BDQ on mitochondrial respiration, do not indicate inhibition of ATP synthesis by ATPase. Inhibition of ATP synthesis normally leads to a decrease in mitochondrial respiration, as is apparent from numerous studies using oligomycin (well-described inhibitor of ATPase) to achieve state 4 respiration [46]. In our experiments, we did not observe reduced respiration rates because of BDQ treatment. In healthy brain mitochondria, administration of BDQ resulted in higher complex I-driven respiration rates including proton leak (CAT_PMG_), ADP-stimulated respiration (ADP_PM_, ADP_PMG_, OXPHOS_PM,_ and OXPHOS_PMG_), and maximal complex I related respiratory capacity (ETC_PMG_). Increased respiration rates in the proton-leak state may be explained by the uncoupling effect of BDQ, which has been observed both in Mycobacteria [47] and in rat liver mitochondria [43]. BDQ has been described as a non-traditional uncoupler that, by binding to ATPase, dissipates the ΔpH across the inner mitochondrial membrane but does not decrease the Δψ_m_ [48]. The decrease in ΔpH reduces the proton motive force needed for ATP synthesis; therefore, uncoupling cannot account for the increase in OXPHOS rate, which is strictly oxidative phosphorylation–associated respiration. Generally, BDQ has been shown to stimulate oxygen consumption both by prokaryotic [49] and eukaryotic cells [50]. Stimulated bacterial respiration is attributed to the uncoupling effect of BDQ because regardless of increased oxygen consumption, ATP synthesis is inhibited [49]. However, a study by Fiorillo et al. showed that cultured human fibroblasts exhibit increased cellular respiration as well as ATP production when exposed to BDQ [50]. This particular observation is in agreement with our results, as we show that BDQ was successful in stimulating phosphorylating respiration (OXPHOS state). It is important to note that in the proton leak-associated respiration, only the CAT_PMG_ rate was increased after BDQ pretreatment. CAT state is achieved by inhibiting adenine nucleotide translocase (ANT), which results in “trapping” ATP inside mitochondria [51]. Increased levels of intra-mitochondrial ATP could possibly result in activation of certain signalling pathways such as phosphorylation of respiratory complexes by protein kinases, thus regulating their activity. In our investigations, respiration with succinate (complex II-driven respiration) was not affected by BDQ, suggesting that BDQ specifically targets NADH-dependent respiration. This was further demonstrated in uncoupled respiration, where BDQ administered to control animals resulted in higher rates of ETC_PMG_ and ETC_MAX_ but not ETC_S_. However, the specific mechanism by which BDQ stimulates OXPHOS respiration rates specifically with NADH-dependent substrates is not entirely clear. 

A statistically significant effect of BDQ treatment after MCAO was observed in the proton leak state CAT_PMG_ and uncoupled state ETC_MAX_. The higher rate of proton leak-linked respiration again demonstrates that mitochondrial respiration was mildly uncoupled by BDQ, while the higher rate in ETC_MAX_ metabolic state indicates that BDQ treatment might limit the ischemia–reperfusion-induced damage to the mitochondrial ETC complexes. Mild uncoupling of mitochondrial respiration has been demonstrated to limit cell death after cardiac ischemia [52] and traumatic brain injury [53]. The protective effect of mild uncoupling is thought to occur because of a reduction in mitochondrial ROS generation. During reperfusion, mitochondria initially become hyperpolarized [15,54], and ROS production is high in this state. BDQ has been shown to have no effect on the Δψ_m_ in mammalian cells [43]; however, it is not clear whether it can reverse the hyperpolarization of mitochondrial membrane following the onset of reperfusion in brain cells. A possible mechanism of neuroprotection by BDQ is preventing this rise in Δψ_m_ along with excessive ROS production. Furthermore, ischemia is reported to cause a decrease in the activities of all 4 ETC complexes [55,56], and this is partly reflected in our study as respiration in the ETC_MAX_ state (where all complexes supposedly work at maximal capacity) was decreased after MCAO. The effect of BDQ in this state is similar to one observed in control animals, again suggesting that BDQ stimulates mitochondrial respiration both in normoxic conditions and during ischemia-reperfusion. 

The direct effects of BDQ on mitochondria isolated from a healthy brain revealed inhibition of ATPase but no effect on the mPTP. Interestingly, only the ATP hydrolysis reaction was inhibited by BDQ. This is partly consistent with the study by Luo et al., in which they show that the IC_50_ value of BDQ for ATP hydrolysis (25 nM) is much lower than the IC_50_ value for ATP synthesis (1.3 µM) in purified yeast F0F1 ATP synthase [39]. For reference, they also measured the inhibition of ATP hydrolysis by oligomycin and found that the IC_50_ of ATP hydrolysis was 100 nM. This comparison shows that ATP hydrolysis is more sensitive to inhibition by BDQ than ATP synthesis, and suggests that BDQ is more effective in inhibiting ATP hydrolysis compared to oligomycin.

ATPase is considered to be the target of BDQ. Experimental studies provide evidence that mPTP component cyclophilin D interacts with ATPase in a cyclosporin A-sensitive manner [57,58]. However, in our experiments, BDQ had no effect on isolated brain mitochondrial calcium retention capacity. Similarly, pretreatment with BDQ had no effect on mitochondrial calcium retention capacity in the MCAO group. These results let us conclude that the protective effect of BDQ against ischemic brain injury is not via mPTP. A study with isolated liver mitochondria showed that preincubation with BDQ inhibits mPTP opening and the release of cytochrome c [43]. Additionally, it should be mentioned that the binding affinity of BDQ to ATP-synthase depends on the surrounding lipid composition and this is one of the explanations why BDQ appears to be species and cell type-specific [39]. 

Our study has some limitations. First, we showed that BDQ was protective against early acute ischemia-induced brain injury. However, it remains unclear whether BDQ can prevent delayed neuronal death, which occurs days and weeks after the onset of ischemia. Second, BDQ in our study was applied as pretreatment before ischemia. Such an approach is unlikely to be possible in most clinical situations. Therefore, it is important to investigate whether BDQ, administered a few hours after an ischemic episode or at the time of reperfusion, will exhibit similar neuroprotective effects. A potential clinical significance of our study can be related to tuberculosis patients who take BDQ. It has been reported that tuberculosis patients have a greater risk of experiencing ischemic stroke compared to the general population, and thus patients taking BDQ might benefit from the neuroprotective effects of this medicine [59].

In conclusion, this study indicates that BDQ is able to alleviate ischemia-reperfusion-induced brain injury in a rodent model of ischemic stroke. Pretreatment with BDQ caused inhibition of ATPase activity, mild uncoupling, and stimulation of the activity of ETC complexes. All of these effects may be components of a possible mechanism of neuroprotection by BDQ. Thus, our findings are important in revealing the mechanisms by which acute neuronal death can be reduced during stroke. Inhibitors of mitochondrial ATPase activity as neuroprotective agents are not extensively studied currently; therefore, our study provides new evidence that this field should be investigated more rigorously.

## 4. Materials and Methods

### 4.1. Animals and Treatment

Experiments were performed on male Wistar rats aged 2–4 months (200–300 g). The rats were housed and handled at the Lithuanian University of Health Sciences animal house in agreement with the Guide for the Care and Use of Laboratory Animals. All experimental procedures were reviewed and approved by the State Food and Veterinary Service of Lithuania (license No. G2-79) according to Directive 2010/63/EU of the European Parliament. The animals were randomly assigned into 4 groups: healthy rats injected with vehicle 24 h before euthanasia (Control group); healthy rats that received 2 mg/kg BDQ intraperitoneally 24 h before euthanasia (Control + BDQ group); rats subjected to MCAO surgery and injected with vehicle 60 min before the onset of ischemia (MCAO group); and MCAO rats injected with 2 mg/kg BDQ 60 min before the onset of ischemia (MCAO+BDQ group). BDQ stock solution was prepared by dissolving BDQ in 10:90 (vol/vol) DMSO:PEG300 mixture and, right before injection, this stock solution was diluted with 60:40 (vol/vol) PEG300:NaCl (0.9%) mixture. BDQ was injected at a dose of 2 mg/kg intraperitoneally 60 min before the onset of ischemia or 24 h before the animal was euthanized. Animals assigned to ischemic groups underwent MCAO surgery, and control animals were not operated on.

### 4.2. Transient Focal Ischemia Model

Animals were anesthetized in an enclosed box using 4% sevoflurane in a 70:30 air and oxygen mixture for induction, and maintained using a nose cone with 3% sevoflurane in the same gas mixture. Prior to operation, animals were administered atropine (0.05 mg/kg intraperitoneally) and lidocaine locally (0.1 mL of 2% solution subcutaneously). Temperature of the animals was maintained at 37 °C throughout the surgical procedure using a heating mat. 

Focal cerebral ischemia was induced using the original Longa et al. MCAO method [60] with slight modifications. Briefly, a midline neck incision was made, and left common carotid artery (CCA) along with left external carotid artery (ECA) and left internal carotid artery (ICA) were exposed. CCA and ICA were gently separated from the adjacent vagus nerve. Small branches of the ECA including the occipital artery and superior thyroid artery were isolated and coagulated. Then, CCA and ECA were permanently ligated using a silk suture, and a microvascular clamp was placed on the ICA as distally as possible. Another silk suture was loosely tied around the CCA proximal to its bifurcation. CCA was punctured with a 25G needle below the loosely tied suture and a special MCAO suture with a silicone tip (Doccol, Sharon, MA, USA) was introduced into the CCA. Microvascular clip from the ICA was removed, and MCAO suture was advanced into the ICA approximately 17–19 mm until resistance was felt, and at this point, the middle cerebral artery (MCA) was considered occluded. To secure the MCAO suture in place, the loosely tied suture on the CCA was tightened. Animals were removed from anesthesia and were allowed to recover. To reintroduce blood flow to the MCA after 60 min of occlusion rats were anesthetized as described previously, the knot proximal to bifurcation of the CCA loosened, and the MCAO suture was removed. The knot was tightened again to prevent bleeding, the incision was closed and sutured, and the rats were administered 5 mg/kg ketoprofen for analgesia subcutaneously and were removed from anesthesia. During recovery for about 1 h, animals were kept in a 37 °C environment and monitored for bleeding and respiratory symptoms. When fully recovered, rats were placed in individual cages with free access to food and water.

### 4.3. Neurological Evaluation

The neurological deficit was evaluated 24 h after the onset of reperfusion according to a modified 5-point neurological impairment scale [60]. Briefly, we tested for neuromuscular function impairment using 4 tests performed in succession: the forelimb extension (flexion) test; torso twisting test; observing for circling to the contralateral side; observing for falling to the contralateral side. Animals that had no neuromuscular deficit were given 0 points and were excluded from the study. Each test was graded by 1 or 0 points according to the behavior of the animal (Table 1).

### 4.4. Evaluation of Infarct Size

Twenty-four hours after the onset of reperfusion, rats were euthanized with increasing concentrations of CO_2_ gas, perfused with ice-cold phosphate-buffered saline (PBS) solution through the heart, and decapitated. Brains were carefully removed and chilled for 5 min in ice-cold PBS. Then cerebella were separated and the remaining brain was sectioned into six 2-mm-thick coronal slices using a brain matrix (AgnTho’s, Lidingö, Sweden). Slices were fully immersed into a 1% 2,3,5-Triphenyl-tetrazolium chloride (TTC) solution and incubated for 20 min in the dark at 37 °C. Images of brain slices were converted to a digital format and analysed using ImageJ software. The surface area of infarct was calculated for all 6 slices on both sides (expressed as % of the surface of the whole slice) and normalized using the Equation (1).

(1) $$Infarct_{norm}=\frac{Infarct_{\;}\times \;S_{contr}}{S_{ips}}$$

*Infarct_norm_* is the normalized infarct size (% of the whole slice); *Infarct* is the measured size of infarct; *S_contr_* is the surface area of the contralateral (non-ischemic) hemisphere and *S_ips_* is the surface area of the ipsilateral (ischemic) hemisphere. The infarct size of one animal is expressed as the average of the normalized infarct sizes from all 12 images.

### 4.5. Isolation of Mitochondria

Animals were euthanized and brains removed as previously described. Mitochondria were isolated only from the left hemisphere. Briefly, the tissue was finely minced using sharp scissors. Then it was homogenized with a glass-Teflon homogenizer in mitochondria isolation buffer (222 mM mannitol, 75 mM sucrose, 5 mM HEPES, 1 mM EGTA, pH 7.4 at 4 °C). The first centrifugation was carried out at 1000× *g* for 5 min at 4 °C, and the supernatant was collected and centrifuged again at 10,000× *g* for 10 min at 4 °C. Pelleted mitochondria were resuspended in a small volume of mitochondria isolation buffer and divided into two portions: one portion was frozen at −80 °C and stored for further experiments, and the remaining part was used for experiments that required freshly isolated mitochondria.

### 4.6. Measurement of Mitochondrial Respiration

**Figure 5:**
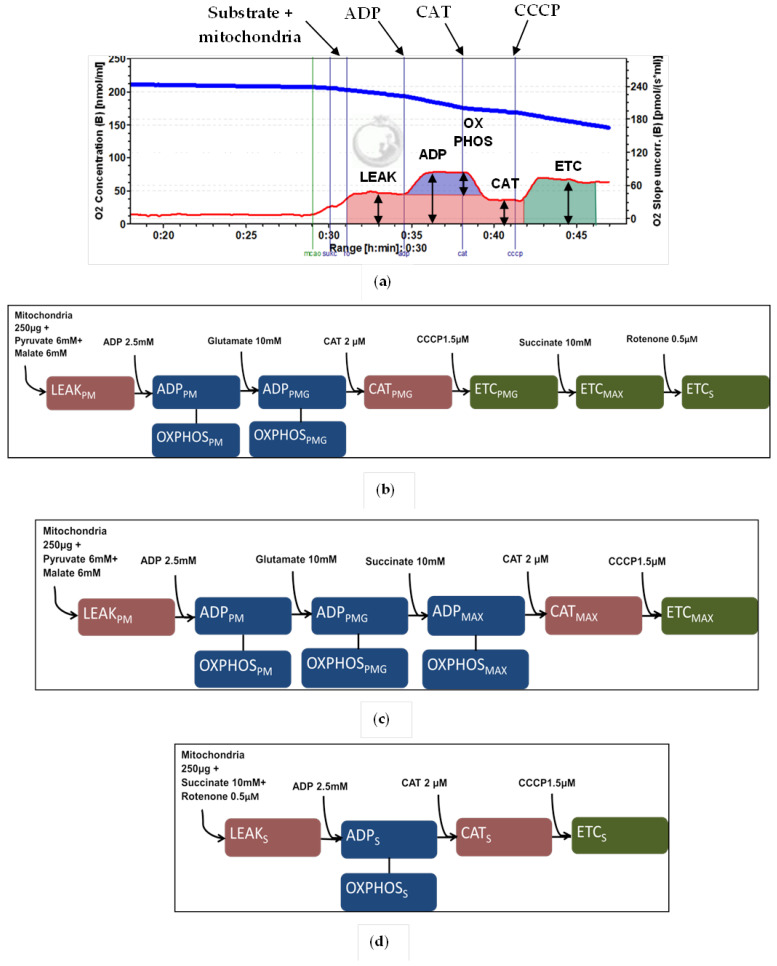
Substrate, uncoupler, and inhibitor titration protocols. (**a**)—Representation of different metabolic states investigated in our experiments. Image is taken from Oroboros DatLab software interface, where the blue line represents oxygen concentration in the chamber and the red line represents the OCRs at a given moment. (**b**)—Substrate, uncoupler, and inhibitor titration protocol 1. (**c**)—Substrate, uncoupler, and inhibitor titration protocol 2. (**d**)—Substrate, uncoupler, and inhibitor titration protocol 3.

Freshly isolated mitochondria were used for respiration measurements. Total mitochondrial protein was measured by the Biuret method. Mitochondrial respiration was recorded by high-resolution respirometry (OROBOROS Oxygraph-2k and Dat-lab software) using 0.125 mg/mL mitochondrial protein at 37 °C. OCRs were recorded in different metabolic states, as illustrated in Figure 5.

Figure 5a shows the distinction between key metabolic states that were recorded during our experiments. Proton leak-linked respiration, highlighted in red, was achieved by adding mitochondria and substrates without ADP (LEAK). After ADP was added to the system, we recorded ADP-stimulated respiration (ADP). OCR, which was exclusively linked to oxidative phosphorylation, was calculated by subtracting the LEAK value from ADP value and termed OXPHOS (highlighted in blue). The addition of CAT after ADP has already been supplied to the system reverts respiration to a state, linked to proton leak, and we termed this metabolic state CAT. Finally, we used an uncoupler, carbonyl cyanide m-chlorophenyl hydrazone (CCCP), to record maximal ETC capacity with a given substrate, and this metabolic state was termed ETC (highlighted in green).

Figure 5b shows titration protocol 1, where pyruvate plus malate (6 + 6 mM) were used as substrates of mitochondrial complex I; after the addition of these substrates and mitochondria (0.125 mg/mL), we recorded OCR, which represented proton leak with these substrates (LEAK_PM_). Then, ADP (2.5 mM) was added, and the rates of ADP_PM_ as well as OXPHOS_PM_ were recorded. Glutamate (10 mM) was added as a complex I substrate that overcomes the pyruvate dehydrogenase complex (PDH) to see whether PDH is a rate-limiting factor in complex I-linked respiration, and rates ADP_PMG_ as well as OXPHOS_PMG_ were recorded. The addition of 2 µM of CAT resulted in a state also representing the proton leak similar to LEAK_PM_, and was termed CAT_PMG_. Next, we added 1.5 µM of CCCP, which resulted in a state of uncoupled respiration, and recorded the maximal ETC capacity with complex I substrates (ETC_PMG_). Succinate (10 mM) was used as a substrate for mitochondrial complex II, and rate ETC_MAX_ was recorded. Rotenone (0.5 µM) was used to inhibit complex I-linked respiration, and the resulting state was considered to be complex II-dependent—ETC_S_ was recorded. Titration protocol 2 is shown in Figure 5c; it contains mostly the same metabolic states as titration protocol 1, except after addition of succinate, maximal ADP-stimulated respiration was achieved, and ADP_MAX_ as well as OXPHOS_MAX_ rates were recorded. Additionally, with this protocol, we recorded respiration linked to proton leak with pyruvate, malate, glutamate, and succinate (CAT_MAX_). Figure 5d represents titration protocol 3, which started with succinate, mitochondria, and rotenone (LEAK_S_ was recorded). Then, the addition of ADP led to ADP_S_ and OXPHOS_S_ states. Addition of CAT resulted in the CAT_S_ metabolic state, and finally, uncoupling with CCCP led to the ETC_S_ state.

In summary, we recorded OCRs in the following metabolic states: proton leak-linked respiration (LEAK_PM_, LEAK_S_, CAT_PMG_, CAT_S_, and CAT_MAX_); ADP-stimulated respiration (ADP_PM_, ADP_PMG_, ADP_S_, and ADP_MAX_ and calculated rates OXPHOS_PM_, OXPHOS_PMG_, OXPHOS_S,_ and OXPHOS_MAX_), and uncoupled respiration (ETC_PMG_, ETC_S_, and ETC_MAX_).

### 4.7. Measurement of Mitochondrial Calcium Retention Capacity

Mitochondrial calcium retention capacity was measured using freshly isolated mitochondria in a medium containing 1 mM KH_2_PO_4_, 10 μM EGTA, 10 mM Tris-HCl, and 200 mM sucrose (pH 7.4) supplemented with 1 mM pyruvate and 1 mM malate. The method of CRC assessment was based on the measurement of fluorescence of the Calcium Green 5N dye with spectrofluorometer PerkinElmer LS-55 (excitation at 507 nm, emission at 538 nm). Before each experiment, the medium was supplemented with 150 nM of Calcium Green 5N, and experiments were started by the addition of 0.17 mg/mL mitochondria. Then, 1.6 µM CaCl_2_ pulses were added every 2 min until a large increase in fluorescence signal was recorded. The large increase in fluorescent signal was determined as the opening of the mPTP.

### 4.8. Measurement of the Enzymatic Activity of ATPase

The activity of complex V (ATPase) was measured using a commercial kit from Abcam (ab109714) according to the manufacturer’s instructions. The ATP synthase complex was immunocaptured within the wells in the microplate. The enzymatic hydrolysis of ATP is coupled to oxidation of NADH in this particular kit; therefore, the enzymatic activity of ATP synthase was recorded by measuring the decrease in absorbance at 340 nm (Infinite 200 PRO microplate reader, Tecan, Austria). The enzymatic activity of ATPase was expressed as the decrease in absorbance at 340 nm per min per 1 mg of mitochondrial protein (ΔOD_340 nm_/min/mg protein).

### 4.9. Statistical Analysis

Statistical analyses were performed using IBM SPSS statistics 22 software. Graphs were made using Sigma plot 13.0 software. Data are presented as means ± 1 standard error. One-way ANOVA with LSD post hoc statistical tests were used to compare means. The difference was considered significant when *p* < 0.05.

## Figures and Tables

**Table 1:** Interpretation of the neurological impairment test. Points were given if the animal failed to extend both forelimbs and/or showed flexion of the contralateral forelimb when held above a flat surface (1 point); animal held by its tail showed twisting of the torso to the ipsilateral side (+1 point); animal showed a tendency to circle to the contralateral side when allowed to freely move (+1 point); and was falling to the contralateral side when allowed to freely move (+1 point).

Test	Neurological Deficit	Score
Forelimb extension (flexion)	Mild	1
Twisting of the torso	Mild-moderate	2
Circling to the right	Moderate	3
Falling to the right	Severe	4

## Data Availability

The data generated and presented in this study are available on request from the corresponding author.

## References

[1] Anderson C.S. (2019). The global burden of stroke: Persistent and disabling. Lancet Neurol..

[2] Campbell B.C., Silva D.A.D., Macleod M.R., Coutts S.B., Schwamm L.H., Davis S.M., Donnan G.A. (2019). Ischaemic stroke. Nat. Rev. Dis. Prim..

[3] Grefkes C., Fink G.R. (2020). Recovery from stroke: Current concepts and future perspectives. Neurol. Res. Pract..

[4] Goyal M., Menon B.K., Zwam W.H.V., Dippel D.W.J., Mitchell P.J., Demchuk A.M., Dávalos A., Majoie C.B.L.M., Berg L.A.V.D., Levy E.I. (2016). Endovascular thrombectomy after large-vessel ischaemic stroke: A meta-analysis of individual patient data from fi ve randomised trials. Lancet.

[5] Fiehler J., Ford I., Galinovic I., Gellissen S., Golsari A., Gregori J., Günther M., Guibernau J., Häusler K.G., Hennerici M. (2018). MRI-Guided Thrombolysis for Stroke with Unknown Time of Onset. N. Engl. J. Med..

[6] Whiteley W.N., Emberson J., Lees K.R., Blackwell L., Albers G., Bluhmki E., Brott T., Cohen G. (2016). Risk of intracerebral haemorrhage with alteplase after acute ischaemic stroke: A secondary analysis of an individual patient data meta-analysis. Lancet.

[7] Behme D., Gondecki L., Fiethen S. (2014). Complications of mechanical thrombectomy for acute ischemic stroke—A retrospective single-center study of 176 consecutive cases. Neuroradiology.

[8] Warach S., Latour L.L. (2004). Evidence of Reperfusion Injury, Exacerbated by Thrombolytic Therapy, in Human Focal Brain Ischemia Using a Novel Imaging Marker of Early Blood-Brain Barrier Disruption. Stroke.

[9] Zhang R., Chopp M., Chen H., Garcia J.H. (1994). Temporal profile of ischemic tissue damage, neutrophil response, and vascular plugging following permanent and transient (2H) middle cerebral artery occlusion in the rat. J. Neurol. Sci..

[10] Lin L., Wang X., Yu Z. (2018). Ischemia-reperfusion Injury in the Brain: Mechanisms and Potential Therapeutic Strategies. Biochem. Pharmacol..

[11] Andrabi S.S., Parvez S., Tabassum H. (2020). Ischemic stroke and mitochondria: Mechanisms and targets. Protoplasma.

[12] Jordan J., Groot P.W.J.D., Galindo M.F. (2011). Mitochondria: The Headquarters in Ischemia-Induced Neuronal Death. Agents Med. Chem..

[13] Grover G.J., Atwal K.S., Sleph P.G., Wang F., Monshizadegan H., Monticello T., Green D.W., Gary J., Atwal K.S., Sleph P.G. (2004). Excessive ATP hydrolysis in ischemic myocardium by mitochondrial F1F0-ATPase: Effect of selective pharmacological inhibition of mitochondrial ATPase hydrolase activity. Am. J. Physiol. Hear. Circ. Physiol..

[14] Gao Y.Q., Yang W., Karplus M., Biophysique L.D.C. (2005). A Structure-Based Model for the Synthesis and Hydrolysis of ATP by F1-ATPase. Cell.

[15] Iijima T. (2006). Mitochondrial membrane potential and ischemic neuronal death. Neurosci. Res..

[16] Berkich D.A., Salama G., Lanoue K.F. (2003). Mitochondrial membrane potentials in ischemic hearts. Arch. Biochem. Biophys..

[17] Chinopoulos C., Adam-vizi V. (2010). Mitochondria as ATP consumers in cellular pathology. BBA.

[18] Matsuyama S., Xu Q., Velours J., Reed J.C. (1998). The Mitochondrial F0F1-ATPase Proton Pump Is Required for Function of the Proapoptotic Protein Bax in Yeast and Mammalian Cells. Mol. Cell.

[19] Formentini L., Cuezva M. (2013). Mitochondria-Mediated Energy Adaption in Cancer: The H+-ATP Synthase-Geared Switch of Metabolism in Human Tumors. Antioxid. Redox Signal..

[20] Formentini L., Pereira M.P., Sánchez-Cenizo L., Santacatterina F., Lucas J.J., Navarro C., Martínez-Serrano A., Cuezva J.M. (2014). In vivo inhibition of the mitochondrial H+-ATP synthase in neurons promotes metabolic preconditioning. EMBO J..

[21] Long Q., Yang K., Yang Q. (2015). Regulation of mitochondrial ATP synthase in cardiac pathophysiology. Am. J. Cardiovasc. Dis..

[22] Nesci S., Trombetti F., Algieri C., Pagliarani A. (2019). A Therapeutic Role for the F1F0-ATP Synthase. SLAS Discov..

[23] Bosetti F., Yu G., Zucchi R., Solaini G. (2000). Myocardial ischemic preconditioning and mitochondrial F1F0-ATPase activity. Mol. Cell. Biochem..

[24] Matic I., Cocco S., Ferraina C., Martin-jimenez R., Florenzano F., Crosby J., Lupi R., Amadoro G., Russell C., Pignataro G. (2016). Neuroprotective coordination of cell mitophagy by the F1F0-ATPase inhibitory factor 1 (IF1). Pharmacol. Res..

[25] Maierean S., Serban M., Rizzo M., Lippi G., Sahebkar A. (2017). The potential role of mitochondrial ATP synthase inhibitory factor 1 (IF1) in coronary heart disease: A literature review. Lipids Health Dis..

[26] Huang L., Chuang I., Dong H., Yang R. (2011). Hypoxia-Inducible Factor 1a Regulates the Expression of the Mitochondrial ATPase Inhibitor Protein (IF1) in Rat Liver. Shock.

[27] Bernardi P., Rasola A., Forte M., Lippe G. (2015). The Mitochondrial Permeability Transition Pore: Channel Formation by F-ATP Synthase, Integration in Signal Transduction, and Role in Pathophysiology. Physiol. Rev..

[28] Bonora M., Wieckowski M.R., Chinopoulos C., Kepp O., Kroemer G., Galluzzi L., Pinton P. (2015). Molecular mechanisms of cell death: Central implication of ATP synthase in mitochondrial permeability transition This article has been corrected since Advance Online Publication and a corrigendum is also printed in this issue. Oncogene.

[29] Hurst S., Hoek J., Sheu S.-S. (2018). Mitochondrial Ca2+ and Regulation of the Permeability Transition Pore. J. Bioenerg. Biomembr..

[30] Li B., Chauvin C., De Paulis D., De Oliveira F., Gharib A., Vial G., Lablanche S., Leverve X., Bernardi P., Ovize M. (2012). Inhibition of complex I regulates the mitochondrial permeability transition through a phosphate-sensitive inhibitory site masked by cyclophilin D. BBA.

[31] Urbani A., Giorgio V., Carrer A., Franchin C., Arrigoni G., Jiko C., Abe K., Maeda S., Shinzawa-itoh K., Bogers J.F.M. (2019). Purified F-ATP synthase forms a Ca2+-dependent high-conductance channel matching the mitochondrial permeability transition pore. Nat. Commun..

[32] Neginskaya M.A., Solesio M.E., Berezhnaya E.V., Amodeo G.F., Mnatsakanyan N., Jonas E.A., Pavlov E.V. (2019). ATP Synthase C-Subunit-Deficient Mitochondria Have a Small Cyclosporine A-Sensitive Channel, but Lack the Permeability Transition Pore. Cell Rep..

[33] Giorgio V., von Stockum S., Antoniel M., Fabbro A., Fogolari F., Forte M., Glick G.D., Petronilli V., Zoratti M., Szabo I. (2013). Dimers of mitochondrial ATP synthase form the permeability transition pore. Proc. Natl. Acad. Sci. USA.

[34] Worley M.V., Estrada S.J. (2014). Bedaquiline: A Novel Antitubercular Agent for the Treatment of Multidrug-Resistant Tuberculosis. Pharmacotherapy.

[35] Haagsma A.C., Podasca I., Koul A., Andries K., Guillemont J., Lill H., Bald D. (2011). Probing the Interaction of the Diarylquinoline TMC207 with Its Target Mycobacterial ATP Synthase. PLoS ONE.

[36] Koul A., Vranckx L., Dendouga N., Balemans W., Van Den Wyngaert I., Vergauwen K., Go H.W.H., Willebrords R., Poncelet A., Guillemont J. (2008). Diarylquinolines Are Bactericidal for Dormant Mycobacteria as a Result of Disturbed ATP Homeostasis. J. Biol. Chem..

[37] Haagsma A.C., Abdillahi-ibrahim R., Wagner M.J., Krab K., Vergauwen K., Guillemont J., Andries K., Lill H., Koul A., Bald D. (2009). Selectivity of TMC207 towards Mycobacterial ATP Synthase Compared with That towards the Eukaryotic Homologue. Antimicrob. Agents Chemother..

[38] Andries K., Verhasselt P., Guillemont J., Gohlmann H.W.H., Neefs J., Winkler H., Van Gestel J., Timmerman P., Zhu M., Lee E. (2005). A Diarylquinoline Drug Active on the ATP Synthase of Mycobacterium tuberculosis. Science.

[39] Luo M., Zhou W., Patel H., Srivastava A.P., Symersky J., Bonar M.M., Faraldo-gómez J.D., Liao M., Mueller D.M. (2020). Bedaquiline inhibits the yeast and human mitochondrial ATP synthases. Commun. Biol..

[40] Shchepina L.A., Pletjushkina O.Y., Avetisyan A.V., Bakeeva L.E., Fetisova E.K., Izyumov D.S., Saprunova V.B., Vyssokikh M.Y., Chernyak B.V., Skulachev V.P. (2002). Oligomycin, inhibitor of the F0 part of H+-ATP-synthase, suppresses the TNF-induced apoptosis. Oncogene.

[41] Jennings R.B., Reimer K.A., Steenbergen C. (1991). Effect of Inhibition of the Mitochondrial ATPase on Net Myocardial ATP in Total Ischemia. J. Mol. Cell. Cardiol..

[42] Mnatsakanyan N., Jonas E.A. (2020). ATP synthase c-subunit ring as the channel of mitochondrial permeability transition: Regulator of metabolism in development and degeneration. J. Mol. Cell. Cardiol..

[43] Belosludtsev K.N., Belosludtseva N.V., Talanov E.Y., Tenkov K.S., Starinets V.S., Agafonov A.V., Pavlik L.L., Dubinin M. (2019). V Effect of bedaquiline on the functions of rat liver mitochondria. Biochim. Biophys. Acta Biomembr..

[44] Pamreddy A., Baijnath S., Naicker T., Ntshangase S., Mdanda S., Lubanyana H., Kruger H.G., Govender T. (2018). Bedaquiline has potential for targeting tuberculosis reservoirs in the central nervous system. RSC Adv..

[45] Penna C., Pagliaro P., Rastaldo R., Di Pancrazio F., Lippe G., Gattullo D., Mancardi D., Samaja M., Losano G., Mavelli I. (2004). F0F1 ATP synthase activity is differently modulated by coronary reactive hyperemia before and after ischemic preconditioning in the goat. Am. J. Physiol. Hear. Circ. Physiol..

[46] Djafarzadeh S., Jakob S.M. (2017). High-resolution respirometry to assess mitochondrial function in permeabilized and intact cells. J. Vis. Exp..

[47] Hards K., Robson J.R., Berney M., Shaw L., Bald D., Koul A., Andries K., Cook G.M. (2015). Bactericidal mode of action of bedaquiline. J. Antimicrob. Chemother..

[48] Nath S. (2019). Interpretation of the mechanism of action of antituberculosis drug bedaquiline based on a novel two-ion theory of energy coupling in ATP synthesis. Bioeng. Transl. Med..

[49] Hards K., McMillan D.G.G., Schurig-Briccio L.A., Gennis R.B., Lill H., Bald D., Cook G.M. (2018). Ionophoric effects of the antitubercular drug bedaquiline. Proc. Natl. Acad. Sci. USA.

[50] Fiorillo M., Lamb R., Tanowitz H.B., Cappello A.R., Martinez-Outschoorn U.E., Sotgia F., Lisanti M.P. (2016). Bedaquiline, an FDA-approved antibiotic, inhibits mitochondrial function and potently blocks the proliferative expansion of stem-like cancer cells (CSCs). Aging (Albany N. Y.).

[51] Klingenberg M. (2008). The ADP and ATP transport in mitochondria and its carrier. Biochim. Biophys. Acta Biomembr..

[52] Brennan J.P., Southworth R., Medina R.A., Davidson S.M., Duchen M.R., Shattock M.J. (2006). Mitochondrial uncoupling, with low concentration FCCP, induces ROS-dependent cardioprotection independent of KATP channel activation. Cardiovasc. Res..

[53] Pandya J.D., Pauly J.R., Sullivan P.G. (2009). The optimal dosage and window of opportunity to maintain mitochondrial homeostasis following traumatic brain injury using the uncoupler FCCP. Exp. Neurol..

[54] Hüttemann M., Helling S., Sanderson T.H., Sinkler C., Samavati L., Mahapatra G., Varughese A., Lu G., Liu J., Ramzan R. (2012). Regulation of mitochondrial respiration and apoptosis through cell signaling: Cytochrome c oxidase and cytochrome c in ischemia/reperfusion injury and inflammation. Biochim. Biophys. Acta Bioenerg..

[55] Racay P., Tatarkova Z., Chomova M., Hatok J., Kaplan P., Dobrota D. (2009). Mitochondrial calcium transport and mitochondrial dysfunction after global brain ischemia in rat hippocampus. Neurochem. Res..

[56] Christensen T., Diemer N.H. (2003). Reduction of Mitochondrial Electron Transport Complex Activity is Restricted to the Ischemic Focus after Transient Focal Cerebral Ischemia in Rats: A Histochemical Volumetric Analysis. Neurochem. Res..

[57] Giorgio V., Bisetto E., Soriano M.E., Dabbeni-Sala F., Basso E., Petronilli V., Forte M.A., Bernardi P., Lippe G. (2009). Cyclophilin D modulates mitochondrial F0F1-ATP synthase by interacting with the lateral stalk of the complex. J. Biol. Chem..

[58] Giorgio V., Fogolari F., Lippe G., Bernardi P. (2019). OSCP subunit of mitochondrial ATP synthase: Role in regulation of enzyme function and of its transition to a pore. Br. J. Pharmacol..

[59] Sheu J.J., Chiou H.Y., Kang J.H., Chen Y.H., Lin H.C. (2010). Tuberculosis and the risk of ischemic stroke: A 3-year follow-up study. Stroke.

[60] Longa E.Z., Weinstein P.R., Carlson S., Cummins R. (1989). Reversible middle cerebral artery occlusion without craniectomy in rats. Stroke.

